# Prediction Model of Aryl Hydrocarbon Receptor Activation by a Novel QSAR Approach, DeepSnap–Deep Learning

**DOI:** 10.3390/molecules25061317

**Published:** 2020-03-13

**Authors:** Yasunari Matsuzaka, Takuomi Hosaka, Anna Ogaito, Kouichi Yoshinari, Yoshihiro Uesawa

**Affiliations:** 1 Department of Medical Molecular Informatics, Meiji Pharmaceutical University, 204-8588 Tokyo, Japan; matsuzys@my-pharm.ac.jp; 2 Laboratory of Molecular Toxicology, School of Pharmaceutical Sciences, University of Shizuoka, Shizuoka 422-8529, Japan; hosaka@u-shizuoka-ken.ac.jp (T.H.); m14018@u-shizuoka-ken.ac.jp (A.O.); yoshinari@u-shizuoka-ken.ac.jp (K.Y.)

**Keywords:** chemical structure, aryl hydrocarbon receptor, DeepSnap, deep learning, QSAR, machine learning

## Abstract

The aryl hydrocarbon receptor (AhR) is a ligand-dependent transcription factor that senses environmental exogenous and endogenous ligands or xenobiotic chemicals. In particular, exposure of the liver to environmental metabolism-disrupting chemicals contributes to the development and propagation of steatosis and hepatotoxicity. However, the mechanisms for AhR-induced hepatotoxicity and tumor propagation in the liver remain to be revealed, due to the wide variety of AhR ligands. Recently, quantitative structure–activity relationship (QSAR) analysis using deep neural network (DNN) has shown superior performance for the prediction of chemical compounds. Therefore, this study proposes a novel QSAR analysis using deep learning (DL), called the DeepSnap–DL method, to construct prediction models of chemical activation of AhR. Compared with conventional machine learning (ML) techniques, such as the random forest, XGBoost, LightGBM, and CatBoost, the proposed method achieves high-performance prediction of AhR activation. Thus, the DeepSnap–DL method may be considered a useful tool for achieving high-throughput in silico evaluation of AhR-induced hepatotoxicity.

## 1. Introduction

Exposure to environmental metabolism-disrupting chemicals (MDCs) contributes to the development and propagation of liver steatosis and hepatotoxicity [1,2,3,4,5,6,7]. The liver is one of the organs most susceptible to drug toxicity, as evidenced from the fact that drug-induced liver injury (DILI) accounts for more than 50% of acute liver failure in humans [8,9]. However, the mechanisms of hepatotoxicity or the adverse outcome pathway (AOP) and health risks remain unknown. Further, discrepancy exists between the results of hepatotoxicity test using animal models and human outcomes [10,11]. The relationship between MDC exposure and the adverse outcome (AO) for hepatotoxicity is often difficult to define because of low-dose effects and non-monotonic dose response [4,12]. The aryl hydrocarbon receptor (AhR; NCBI gene ID: 25690) is a member of the family of ligand-dependent basic helix–loop–helix transcription factors that sense environmental exogenous and endogenous ligands or xenobiotic chemicals, including kynurenine, flavonoids, polyphenols, indoles, halogenated aromatic hydrocarbons, halogenated polycyclic aromatic hydrocarbons, and dioxin-like compounds. To date, more than 400 AhR ligands have been identified which regulate the expression genes involved in diverse biological functions, including detoxification of enzymes, immunity, cell proliferation and differentiation, apoptosis, migration, adhesion, and stem cell maintenance [13,14,15,16,17]. In the canonical AhR signaling pathway, cytosolic AhR translocates into the nucleus upon binding of the ligand, where it dimerizes with the aryl hydrocarbon receptor nuclear translocator (ARNT; NCBI gene ID: 25242), Then, the protein dimer binds directly or indirectly with DNA consensus sequence elements (termed AhR-, dioxin-, or xenobiotic-response elements: AhRE, DRE, or XRE), containing the core sequence 5’-GCGTG-3’, located in the 5’-regulatory region of dioxin-response genes, including cytochrome P4501A1 (*CYP1A1*), *CYP1B1*, the aryl hydrocarbon receptor repressor (*AHRR*; NCBI gene ID: 498999), indoleamine 2,3-dioxygenase 1 (*Ido1*; NCBI gene ID: 66029), and nuclear factors, erythoid 2-like 2 (*Nfe2l2*; NCBI gene ID: 83619), in an asymmetric manner [18,19,20,21,22,23]. The activation of the AhR signal pathway by the cellular responses against environmental toxins and carcinogens elicits hepatotoxicity and tumor propagation in liver [24,25,26]. In particular, 2,3,7,8-tetrachlorodibenzo-*p*-dioxin (TCDD) has been shown to be effectively promote liver tumor by binding with AhR [26,27]. In addition, AhR has been shown to regulate liver polyploidization via phosphoinositide 3-kinase (PI3K), extracellular signal-regulated kinase (ERK), and Wnt/beta-catenin signaling [28]. However, the mechanisms underlying AhR-induced hepatotoxicity and tumor propagation in the liver await complete revelation, and the role of the AhR pathway against the AO and DILI is controversial. Therefore, identification of modulators in AhR signaling and prediction of the mechanical relationship between these modulators and hepatocarcinogenesis are extremely pivotal issues. 

The two-dimensional quantitative structure–activity relationship (2D-QSAR) method has been applied to build prediction models of toxicity by determining the physical and chemical properties of chemical compounds from their chemical structures [29,30,31,32,33]. However, in conventional QSAR analysis, there are some problems concerning limited prediction performance [34,35,36,37]. Recently, QSAR analysis using the deep neural network (DNN) has shown superior prediction performance compared with other conventional machine learning (ML) methods [38,39,40,41,42]. Such high-performance prediction methods may rely on the clear definition of feature representation or selection as it depends on the chemical space [43,44]. For appropriate feature selection or representation, some exclusive procedures based on chemical intuition and observed properties or filtering methods that evaluate features according to a given criterion have been employed [43,45]. However, these approaches do not completely apply to the construction of all prediction models because of the complicated interactions of multiple molecular descriptors. Therefore, a novel description tool of chemical compounds was developed, called DeepSnap [46]. DeepSnap can depict the steric conformation of a chemical structure as a ball-and-stick model, and images can be automatically generated based on the viewing directions along the x-, y-, and z-axes [46]. By using the resulting image data as input, a prediction model can be classified and constructed by deep learning (DL); thus, we refer to the method as DeepSnap–DL. In addition, we recently reported that the high performance of prediction models of molecular initiating event (MIE) activity for the AOP can be constructed by optimizing hyperparameters and adjusting input data preparation [47,48,49].

In this study, the DeepSnap–DL method was used to construct the prediction model of AhR activation using information related to 201 chemical compounds obtained from commercial sources. Using AhR-responsive reporter gene, containing three repeats of the xenobiotic-response element, AhR-responsive activation by a total of 201 chemicals was determined in vitro. Out of three fold-change values of three kinds of concentrations of test compounds, the highest value was used as the “MAX” value, and its average for total chemicals is 1.53 ± 1.50. 

The prediction performance of the AhR model was examined in terms of the snapshot angle, input data split, and MAX value of AhR activation. The proposed prediction model of AhR activation achieved AUC, BAC, and MCC values of 0.959 ± 0.025, 0.933 ± 0.040, and 0.845 ± 0.075, respectively. These findings suggest that a high-performance prediction model can be built using appropriate data preparation and parameter optimization, even with a small input data size.

## 2. Results and Discussion

### 2.1. Optimization of Hyperparameters in the DeepSnap–DL Approach

We have previously shown that hyperparameters in the DeepSnap–DL process affect the performance of prediction models [47,48,49]. Therefore, three main hyperparameters—solver types (STs), batch sizes (BTs), and learning rates (LRs)—in the DeepSnap–DL process were preliminarily optimized using the input data of 201 chemical compound structures. The data were randomly divided into training (Tra), validation (Val), and test (Test) datasets in 1:1:1 ratio; Loss (Val), which is an error rate between the results obtained from the validation data and its labeled dataset; and Acc (Val), which is the percentage of correct answers based on the results obtained from the validation dataset and its labeled dataset and were considered as indicators of prediction performance. Among the six STs―NAG, AdaGrad, AdaDelta, Adam, RMSprop, and SDG―examined at a snapshot angle of 176° for BT:40 and LR:0.001, the lowest Loss (Val) (0.2592) and the highest Acc (Val) (87.70) were observed in NAG and RMSprop (Table S1a). Furthermore, we assessed eight LRs (from 0.0018 to 0.0025) and six BSs (from 35 to 40) at a snapshot angle of 176°. In this study, the highest prediction performance was achieved in NAG of the ST, with Loss (Val) and Acc (Val) being 0.1466 and 94.08, respectively, for LR:0.0025 and BS:37 (Table S1b,c). Therefore, we used the following hyperparameters in the subsequent analysis: ST:NAG, LR:0.0025, and BS:37. 

### 2.2. Snapshot Angles and Input Data Split of Chemical Compounds in the DeepSnap–DL Approach

**Figure 1:**
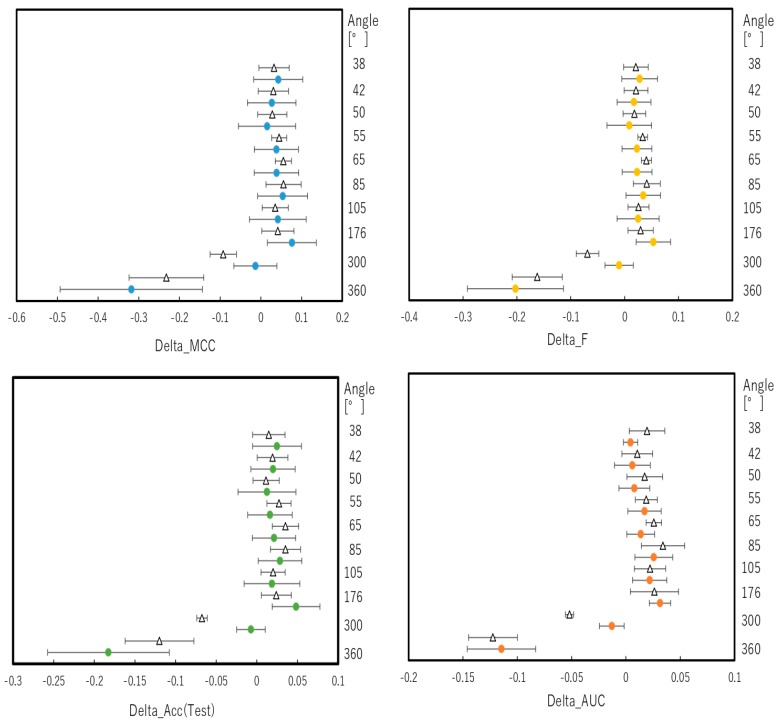
Difference in the mean levels of performance of DeepSnap at different angles. Differences in the mean values of the performance indicators between one angle and the other nine angles, denoted as Delta_MCC, Delta_Acc (Test), Delta_F, and Delta_AUC, were calculated based on the results shown in Figure 1, with a 95% confident interval (CI) as error bars. The filled dots and unfilled triangles indicate the dataset ratios Tra/Val/Test = 1:1:1 and 2:2:1, respectively.

To predict the performance of the DeepSnap approach in detail, we investigated the contribution of the angle at which the Jmol-generated images are captured. For this purpose, we optimized 10 angles with respect to the x-, y-, and z-axes from (360°, 360°, 360°) to (38°, 38°, 38°) using two datasets of 201 chemical compounds divided into Tra/Val/Test = 1:1:1 and 2:2:1. The number of images produced from the three-dimensional (3D) chemical structures using the DeepSnap approach at different angles with respect to the x-, y-, and z-axes was as follows: (360°, 360°, 360°)—1 image, (300°, 300°, 300°)—8 images, (176°, 176°, 176°)—27 images, (105°, 105°, 105°)—64 images, (85°, 85°, 85°)—125 images, (65°, 65°, 65°)—216 images, (55°, 55°, 55°)—343 images, (50°, 50°, 50°)—512 images, (42°, 42°, 42°)—729 images, and (38°, 38°, 38°)—1000 images. The highest prediction performance was achieved at an angle of 176° with a significant difference from the performance at other angles for the dataset ratio Tra/Val/Test = 2:2:1. The values of mean MCC, Acc (Test), AUC, Loss (Val), Acc (Val), F, and BAC at LR: 0.01 were 0.689 ± 0.173, 0.910 ± 0.079, 0.959 ± 0.043, 0.016 ± 0.104, 99.72 ± 3.64, 0.705 ± 0.168, and 0.911 ± 0.063, respectively (Figure S1). However, these results might depend on the initial molecular conformation. Therefore, an average of ten angles for each evaluation index were calculated. At the dataset ratio Tra/Val/Test = 1:1:1, the values of mean MCC, Acc (Test), AUC, Loss (Val), Acc (Val), F, and BAC were 0.634 ± 0.136, 0.820 ± 0.070, 0.884 ± 0.064, 0.379 ± 0.083, 82.43 ± 4.51, 0.721 ± 0.095, and 0.852 ± 0.070, respectively. At the dataset ratio Tra/Val/Test = 2:2:1, the values of mean MCC, Acc (Test), AUC, Loss (Val), Acc (Val), F, and BAC were 0.664 ± 0.162, 0.828 ± 0.087, 0.886 ± 0.058, 0.345 ± 0.088, 84.08 ± 4.08, 0.756 ± 0.100, and 0.860 ± 0.086, respectively. Although we cannot rule out random effects, it has been shown that conformational changes of molecules, which are the position and shape of key functional groups, affect the biological activity [50,51]. Further, it was reported that the energy profile in a fragment of chemical changes periodically with torsion angles [51]. Given these reports, the depiction angles in DeepSnap may play an important role in efficient feature extraction. By contrast, for the dataset ratio Tra/Val/Test = 1:1:1, the prediction performance at an angle of 85° was significantly higher compared with that at the other angles; the values of mean MCC, Acc (Test), AUC, Loss (Val), Acc (Val), F, and BAC at LR: 0.01 were 0.684 ± 0.007, 0.852 ± 0.049, 0.915 ± 0.034, 0.365 ± 0.064, 81.96 ± 3.68, 0.758 ± 0.057, and 0.875 ± 0.033, respectively (Figure S1). In addition, the five performance indicators, namely MCC, Acc (Test), AUC, F, and BAC, at 300 and 360° for Tra/Val/Test = 1:1:1 and at 360° for Tra/Val/Test = 2:2:1 were significantly lower compared with those at the remaining angles (Figure S1). Next, the difference distribution of the mean values of the performance indicators among the 10 angles indicates that the means of MCC, Acc (Test), AUC, F, and BAC at 360° for Tra/Val/Test = 1:1:1 and 2:2:1 datasets were considerably lower compared with those at the remaining nine angles (Figure 1 and Figure S2). These results suggest that in using multiple images as input data, compared to a single image, useful features for modeling may be extracted due to an increase in the amount of information on the chemical structures. 

### 2.3. Contribution of Threshold of AhR Activation in Prediction Performance of the DeepSnap–DL Approach

**Figure 2:**
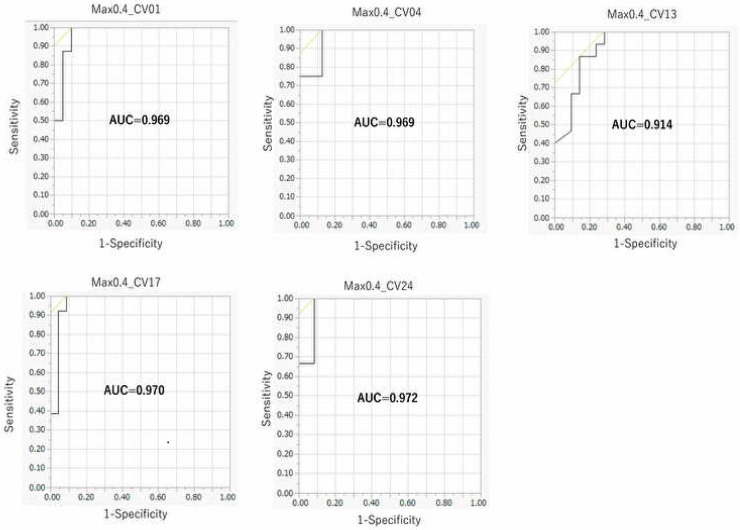
ROC of prediction models in the ≥ 40% threshold. CV01_MAX0.3, CV04_MAX0.3, CV13_MAX0.3, CV17_MAX0.3, and CV24_MAX0.3 represent each dataset in five-fold test.

Next, to examine the effect of the threshold of AhR activation on the prediction performance of the DeepSnap–DL approach, we classified the datasets of 201 chemical compounds according to nine thresholds of MAX values of AhR activation as follows: top ≥ 10%, ≥ 15%, ≥ 20%, ≥ 30%, ≥ 35%, ≥ 40%, ≥ 45%, ≥ 50%, and ≥ 55%. These datasets were divided into Tra/Val/Test = 2:2:1. The results indicated the highest prediction performance at 176°. The mean MCC, AUC, and BAC for the ≥ 40% threshold were 0.845 ± 0.075, 0.959 ± 0.025, and 0.933 ± 0.040, respectively; the mean Acc (Test) for the ≥ 30% threshold was 0.940 ± 0.032; and the mean Loss (Val), Acc (Val), and F for the ≥ 50% threshold were 0.142 ± 0.063, 95.56 ± 2.46, and 0.914 ± 0.027, respectively (Table 1 and Table S2, Figure 2). 

To investigate the effect of dataset split on the prediction performance, a permutation test was conducted in which AhR activation labels that randomly jumbled on the ≥ 40% threshold through five-fold. The result showed that the mean MCC, Acc (Test), AUC, Loss (Val), Acc (Val), F, and BAC at LR: 0.01 were 0.216± 0.254, 0.615 ± 0.093, 0.539 ± 0.064, 0.666 ± 0.013, 61.43 ± 4.67, 0.469 ± 0.215, and 0.594 ± 0.107, respectively (Table 1 and Table S2). 

The effect of the class imbalance problem on classification performance of CNN remains to be addressed in ML. It was reported that the effect of class imbalance on classification performance is detrimental, and thresholding that compensates for prior class probabilities and oversampling to completely eliminate the imbalance dataset could be applied [50]. Further, the oversampling does not cause overfitting of CNNs [50]. In this study, we observed high classification performance at class balanced datasets compared with class imbalanced datasets. These findings suggest that there is possibility of improvement of classification performance in the DeepSnap–DL method by optimization of balance of input data class.

### 2.4. Comparison between the DeepSnap–DL Approach and Four Conventional MLs

**Figure 3:**
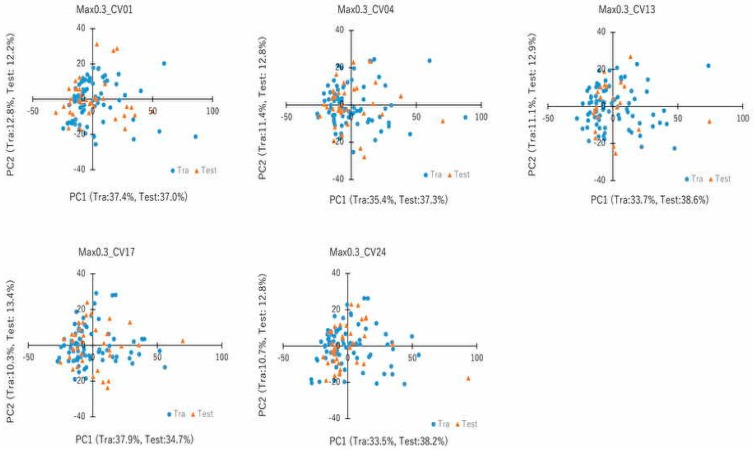
PCA of molecular descriptors. PC1 and PC2 indicate principal components 1 and 2, respectively. Tra and Test datasets are shown by blue dots and orange triangles, respectively. Five datasets with the threshold of top ≥ 30% are denoted as MAX0.3_CV01, MAX0.3_CV04, MAX0.3_CV13, MAX0.3_C17, and MAX0.3_CV24.

To compare the performance of the DeepSnap–DL approach with the other conventional ML techniques, we applied random forest (RF), eXtreme gradient boosting (XGB), light gradient boosting machine (LGBM), and CatBoost (CB) to construct prediction models with 1221 molecular descriptors extracted by open-source software application, Mordred, from the same Tra and Test datasets used in the DeepSnap–DL analysis. Thirty-six prediction models with nine MAX value, which is maximum fold-change value of AhR reporter activity, thresholds (top ≥ 10%, ≥ 15%, ≥ 20%, ≥ 30%, ≥ 35%, ≥ 40%, ≥ 45%, ≥ 50%, and ≥ 55%) were built using the four MLs with Tra/Test = 2:1. The highest mean AUC (0.802 ± 0.075) was observed for the ≥ 30% threshold by the CB (Table 2). The highest mean Acc (Val) (0.894 ± 0.053) was achieved for the ≥ 10% threshold by the XGB (Table 2). In addition, a permutation test on the ≥ 40% threshold showed that the mean AUC were 0.452 ± 0.105, 0.531 ± 0.066, 0.467 ± 0.031, and 0.467 ± 0.048, and the mean Acc (Val) were 0.522 ± 0.077, 0.578 ± 0.060, 0.489 ± 0.032, and 0.533 ± 0.063 by the RF, XGB, LGBM, and CB, respectively (Table 2). Physicochemical and chemical structural properties of small chemical compounds are pivotal data for ML approaches to assess their bioactivities. The resulting properties are applied to cluster component analysis by similar property profiles and principle component analysis (PCA) for reducing variables in the descriptors to explain most of the variables in the original data and eliminating the redundancy [51,52]. The PCA of the molecular descriptors indicated that the mean eigenvalue of principal component (PC) 1 and PC2—which are the first two PCs for explaining the total information contained in the molecular descriptors—for five of the Tra and Test datasets using the four MLs for the ≥ 30% threshold were PC1 (Tra): 35.6% ± 2.04%, PC1 (Test): 37.2% ± 1.52%, PC2 (Tra): 11.3% ± 0.96%, and PC2 (Test): 12.8% ± 0.43% (Figure 3). 

**Figure 4:**
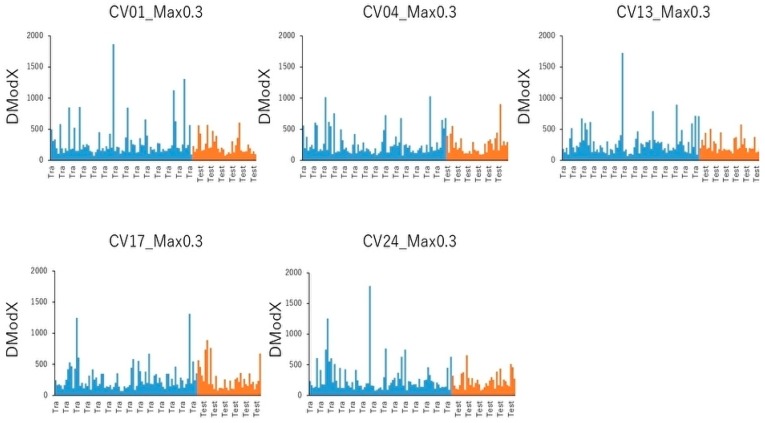
Distance to the model (DmodX) plot. Score scatterplot of the PCA model of data X. Tra and Test datasets were shown by blue and orange bars, respectively. Five datasets on top ≥ 30% of threshold were exhibited as CV01_Max0.3, CV04_Max0.3, CV13_Max0.3, CV17_Max0.3, and CV24_Max0.3.

To group similar variables, identify the most representative variable in each grouping, and calculate the total variation in the predictors explained by these most representative variables, cluster analysis of PC1 and PC2 was performed by five of the Tra and Test datasets. The top 10 cluster ranking for fluctuation in cluster, which is the percentage of fluctuation explained by the PCs of the fluctuations in variables belonging to a cluster, is listed in Table S3a,b. The means of the percentage of total variation explained by the top 10 cluster components in the five-fold validation of Tra and Test datasets were 4.42% ± 1.61% (Tra) and 3.68% ± 0.31% (Test), respectively (Table S3a,b). In addition, to assess the distance of given observation to the PC model plane, Dmodx (distance to the model in X space) was calculated in the five of Tra and Test datasets (Figure 4). As indicated in Figure 4, there were some different moderate outliers in the five Tra datasets, while in the Test datasets, most observations were quite close in values. In addition, the five datasets showed no significant differences of the Dmodx value between the Tra and Test datasets.

## 3. Conclusions

In this study, we constructed prediction models of AhR activation using a DeepSnap–DL approach. As advantages of this approach, first, features in an image can be extracted automatically by CNN without feature selection. Second, information of 3D chemical structure can be used as input data into DL by production of various images with different angles, unlike for a graph structure. Third, high prediction performance can be expected when using DL with CNN. On the other hand, stress on the novel method is a relatively high calculation cost. Further, the identification of portions of features in the image is unclear. The results of the experiments conducted using the proposed method indicated that it achieved a higher performance compared with other conventional MLs, even with a relatively small size of input data. These findings suggest that the DeepSnap–DL method may be a useful tool that can be applied to achieve high-throughput in silico evaluation of AhR-induced hepatotoxicity. Furthermore, this novel DeepSnap–DL method has potential not only for binary classification analysis but also for regression analysis.

## 4. Materials and Methods

### 4.1. AhR Activation Assay

Test compounds were obtained from commercial sources (information available upon request) and dissolved in dimethyl sulfoxide (FUJIFILM Wako Pure Chemical Corporation, Osaka, Japan), ethanol (FUJIFILM Wako Pure Chemical Corporation), or distilled deionized water (Nippon Gene; Toyama, Japan). The AhR-responsive reporter plasmid, (XRE)_3_-tk-pGL4.10, was constructed as follows: oligonucleotides containing three repeats of the xenobiotic-response element from rat *CYP1A1* promoter, 5′-GTACC(CTCTTCTCACGCAACTC)_3_A-3′ and 5′-GATCT (GAGTTGCGTGAGAAGAG)_3_ G-3′, were annealed and inserted into the *Acc*65I and *Bgl*II sites of tk-pGL4.10 [53]. The *Renilla* luciferase plasmid pGL4.74 was purchased from Promega (Madison, WI, USA). All plasmids for transfection were purified using QIAGEN Plasmid Plus Midi Kit (Qiagen; Venlo, the Netherlands). The rat hepatoma-derived H4IIE cells were obtained from American Type Culture Collection (Manassas, VA, USA) and cultured in Dulbecco’s Modified Eagle Medium (FUJIFILM Wako Pure Chemical Corporation) supplemented with 10% heat-inactivated FBS (GE Healthcare; Little Chalfont, Buckinghamshire, UK), 1% Antibiotic-Antimycotic (Thermo Fisher Scientific; Waltham, MA, USA), and 1% MEM Non-Essential Amino Acids (Thermo Fisher Scientific) at 37 °C in a 5% CO_2_ humidified incubator. H4IIE cells were seeded in 96-well plates at 1.5 × 10^4^ cells/well and were reverse-transfected with (XRE)_3_-tk-pGL4.10 (50 ng/well) and pGL4.74 (50 ng/well) using Lipofectamine 3000 Transfection Reagent (Thermo Fisher Scientific). Twenty-four hours later, the culture media was changed to FBS-free Dulbecco’s Modified Eagle Medium containing vehicle or each test compound at 10, 30, or 100 μM. After 24 h treatment, reporter activity was determined using the Dual-Luciferase Reporter Assay System (Promega) and GloMax Navigator System (Promega). Firefly luciferase activity was normalized to *Renilla* luciferase activity, and the AhR-activating potency of each test compound at each concentration was calculated as fold-change relative to vehicle control. Out of the three fold-change values (corresponding to 10, 30, and 100 μM) of each test compound, the highest value was used as the “MAX” value. 

### 4.2. Data Split by Endpoint

In this study, the original datasets of 201 chemical compound structures were prepared in the simplified molecular input line entry system (SMILES) format (Table S4). The MAX values of AhR activation calculated by AhR reporter assay were defined as endpoint scores. The 201 chemical compounds were grouped into two classes based on nine thresholds of the top 10% and the other 90%, top 15% and the other 85%, top 20% and the other 80%, top 30% and the other 70%, top 35% and the other 65%, top 40% and the other 60%, top 45% and the other 55%, top 50% and the other 50%, and top 55% and the other 45% of the MAX values. 

### 4.3. Preparation of Dataset

As for preparation of dataset split into Tra, Val, and Test, two datasets with ratios of Tra/Val/Test = 1:1:1, and 2:2:1 were prepared. For example, in the split procedure for Tra/Val/Test = 2:2:1, the dataset was first split into five groups. Three dataset groups, including Tra, Val, and Test, were then built with a ratio of 2:2:1. A prediction model was created using the Tra and Val datasets, respectively. Finally, prediction performance was calculated by using the Test dataset (2:2:1_01) (Figure S3). For the next analysis, the other test dataset was selected from the group separate from the first analysis, after which the model was built and its probability calculation was examined in the same manner (2:2:1_02). When the five-times analysis was completed (2:2:1_05), a new five-segment dataset was prepared (2:2:1_06). Similarly, the model was constructed by the Tra and Val datasets, and its performance was evaluated by the Test dataset. Finally, a total of 25 tests were performed (2:2:1_25) (Figure S3).

### 4.4. DeepSnap

We applied a 3D conformation import from the SMILES format using MOE 2018 software (MOLSIS Inc.; Tokyo, Japan) to generate a chemical database with the database washing conditions set to protonation state: neutralize; and coordinating washed species: CORINA classic software (Molecular Networks GmbH, Nürnberg, Germany) [54]. The resulting 3D structures were then saved in an SDF file format [47,48,49]. Using the SDF files prepared by the MOE application, the 3D chemical structures were depicted as 3D ball-and-stick models with different colors corresponding to different atoms by Jmol, an open-source Java viewer software for 3D molecular modeling of chemical structures [46,47,48,49]. This 3D chemical structures produces different images depending on the direction. The 3D chemical models were captured automatically as snapshots with user-defined angle increments with respect to the x-, y-, and z-axes. In this study, 10 angle increments were used: (38, 38, 38), (42, 42, 42), (50, 50, 50), (55, 55, 55), (65, 65, 65), (85, 85, 85), (105, 105, 105), (176, 176, 176), (300, 300, 300), and (360, 360, 360). The snapshots were saved as 256 × 256 pixel resolution PNG files (RGB) and divided into three types of datasets: training (Tra), validation (Val), and test (Test). The dataset in this study was divided considering two split ratios, namely Tra/Val/Test = 1:1:1 or 2:2:1.

### 4.5. ML Models

We chose five different ML models to construct the prediction model of AhR activation: (1) DL, (2) RF, (3) XGB, (4) LightGBM, and (5) CB [55]. For the DL, all the PNG image files produced by DeepSnap were resized using NVIDIA DL GPU Training System (DIGITS) version 4.0.0 software (NVIDIA, Santa Clara, CA, USA) on four-GPU systems, Tesla-V100-PCIE (31.7GB), with a resolution of 256 × 256 pixels as input data [47,48,49]. We used a pre-trained open-source DL model, Caffe with ILSVRC (ImageNet Large Scale Visual Recognition Challenge) 2012 dataset [56], which included 1000 class names such as animal (40%), device (12%), container (9%), consumer goods (6%), equipment (4%). The data extracted from ImageNet [57] was split into 1.2 million Tra, 50,000 Val, and 1 million Test datasets. In the DIGITS, two CNNs, AlexNet and GoogLeNet can be used for image classification. It has been shown that GoogLeNet performs better than AlexNet for classification, detection, and counting [48,58,59,60,61,62,63]. For training the model, we used GoogLeNet with a 22 layer deep CNN, comprising two convolutional layers, two pooling layers (four MAX pools and one AVG pool), and nine “Inception” modules, in which each module has six convolution layers, one pooling layer, and 4 million parameters [64,65]; the deep CNN was implemented using open-source software on the CentOS Linux distribution 7.3.1611. We performed 25- and 5-fold experiments to investigate the effect of angle increments and dataset split ratios on prediction performance.

For the RF, XGB, LightGBM, and CB, we calculated the molecular descriptors using the same Tra and Test datasets used in DL with the Python package Mordred [66,67]. We conducted classification experiments in the Python programming language using specific classifier implementations for RF [68], XGB [69], LightGBM [70], and CB [71], provided by the scikit-learn and rdkit Python packages [47,48,49,72].

### 4.6. Evaluation of the Predictive Model

Through 25- or 5-time tests on the Test datasets for the experiments on angle increments and dataset split ratios, which are Tra/Val/Test = 1:1:1 and 2:2:1 in the DL prediction model, we analyzed the probability of the prediction results with the lowest minimum Loss (Val) value, which is an error rate between the results obtained from the validation data and its labeled dataset among 30 examined echoes. We calculated the probabilities for each image of one molecule captured at different angles with respect to the (*x*-, *y*-, and *z*-axes) using the DeepSnap–DL method. Therefore, the medians of each of these predicted values were used as the representative values for target molecules [47,48,49]. The performance of each model in predicting AhR activation was evaluated in terms of the metrics ROC_AUC, BAC, and Acc, which is the percentage of correct answers based on the results obtained from the validation dataset and its labeled dataset, F-measure, and MCC calculated using JMP Pro 14, which is a statistical discovery software (SAS Institute Inc.; Cary, NC, USA) [47,48,49]. These performance metrics are defined as follows:

Sensitivity = ΣTPs / (ΣTPs + ΣFNs)
Specificity = ΣTNs / (ΣTNs + ΣFPs)
BAC = (sensitivity + specificity)/2
Accuracy = (TP + TN) / (TP + FP + TN + FN)
Precision = TP / (TP + FP)
Recall = TP / (TP + FN)
F-measure = 2 × Recall × Precision / (Recall + Precision)
MCC = (TP × TN − FP × FN)/ $\sqrt{\left(\mathrm{TP}+\mathrm{FP}\right)\times \left(\mathrm{TP}+\mathrm{FN}\right)\times \left(\mathrm{TN}+\mathrm{FP}\right)\times \left(\mathrm{TN}+\mathrm{FN}\right)}$

where TP, FN, TN, and FP denote true positive, false negative, true negative, and false positive, respectively. As determination of optimal cutoff point for definition of TP, FN, TN, and FP, the method of maximizing of sensitivity − (1 − specificity) that is called the Youden index [73,74]—that has a range from 0 to 1, with the values closer to 1 representing that effectiveness is larger, while the values closer to 0 are limited effectiveness—is adopted using JMP Pro software. The differences in the mean values of AUC, BAC, F, MCC, Loss (Val), Acc (Test), and Acc (Val) between one angle and the other nine angles are indicated as Delta_AUC, Delta_BAC, Delta_F, Delta_MCC, Delta_ Loss (Val), Delta_ Acc (Test), and Delta_ Acc (Val), respectively, with 95% CI as calculated by Microsoft Excel 2016.

For RF, XGB, LGB, and CB, we calculated the AUC using Python 3 and open-source ML libraries, including scikit-learn [47,48,49]. 

### 4.7. PCA and Cluster Analysis

PCA was performed to compare the distribution of molecular descriptors of the Tra and Test datasets by transforming multidimensional data into a reduced-dimensional space of principal components. In this study, the PCA of the molecular descriptors extracted from the chemical compounds of the same Tra and Test datasets used for building the prediction models by the DL was performed using JMP Pro 14 [47,48,49]. DModX which is the observed distance to the PCs model was calculated using JMP Pro 14, and defined as follows:

$$\sqrt{\mathrm{DModX}=\frac{\sum e_{ik}^{2}}{K-A}}$$

where e_ik_ is the residual from the model, K is the number of variables, and A is the number of PCs [75,76,77,78].

### 4.8. Statistical Analysis

Differences in prediction performances in terms of the parameter loss (Val), Acc (Val), BAC, F, AUC, Acc (Test), and MCC were analyzed using the Mann–Whitney U test [79,80,81]. For each of the 10 angles (38, 38, 38), (42, 42, 42), (50, 50, 50), (55, 55, 55), (65, 65, 65), (85, 85, 85), (105, 105, 105), (176, 176, 176), (300, 300, 300), and (360, 360, 360) in the two datasets Tra/Val/Test = 1:1:1 and 2:2:2, seven evaluation indicators of loss (Val), Acc (Val), BAC, F, AUC, Acc (Test), and MCC are represented as box plots. Significant differences are calculated for each angle. Result with *p* < 0.05 were considered statistically significant.

## Figures and Tables

**Table 1:** Performance of prediction models with thresholds of MAX score by DeepSnap-DL.

MAX Scores	Average AUC ± SD			Average Acc (Test) ± SD			Average MCC ± SD		
0.10	0.863	±	0.121	0.867	±	0.099	0.624	±	0.171
0.15	0.793	±	0.111	0.750	±	0.094	0.481	±	0.139
0.20	0.853	±	0.072	0.828	±	0.036	0.622	±	0.083
0.30	0.920	±	0.029	**0.940**	±	0.032	0.814	±	0.024
0.35	0.936	±	0.025	0.889	±	0.039	0.774	±	0.079
0.40	**0.959**	±	0.025	0.922	±	0.036	**0.845**	±	0.075
0.45	0.919	±	0.045	0.883	±	0.046	0.771	±	0.092
0.50	0.953	±	0.016	0.917	±	0.020	0.842	±	0.031
0.55	0.892	±	0.073	0.861	±	0.071	0.733	±	0.127
0.40PMT	0.539	±	0.064	0.615	±	0.093	0.216	±	0.254

Each average and standard deviation (SD) were calculated by 5-fold cross-validation. Most high-performance of prediction in each MAX Scores were indicated by bold. 0.40PMT showed permutation test in 0.40 of MAX Score.

**Table 2:** Performance of prediction models with thresholds of MAX score by MLs.

MAX Scores	MLs	Average AUC ± SD			Average Acc (Test) ± SD		
0.10	RF	0.642	±	0.151	0.878	±	0.037
	XGB	0.720	±	0.113	**0.894**	±	0.053
	LGBM	0.746	±	0.176	0.872	±	0.046
	CB	0.702	±	0.099	0.878	±	0.037
0.15	RF	0.660	±	0.101	0.822	±	0.032
	XGB	0.736	±	0.083	0.833	±	0.062
	LGBM	0.795	±	0.108	0.817	±	0.072
	CB	0.736	±	0.050	0.817	±	0.046
0.20	RF	0.652	±	0.100	0.767	±	0.032
	XGB	0.687	±	0.118	0.744	±	0.050
	LGBM	0.710	±	0.075	0.744	±	0.066
	CB	0.744	±	0.089	0.767	±	0.042
0.30	RF	0.682	±	0.115	0.733	±	0.050
	XGB	0.770	±	0.104	0.756	±	0.099
	LGBM	0.751	±	0.147	0.772	±	0.050
	CB	**0.802**	±	**0.075**	0.767	±	0.075
0.35	RF	0.737	±	0.062	0.744	±	0.087
	XGB	0.743	±	0.057	0.711	±	0.085
	LGBM	0.732	±	0.079	0.733	±	0.093
	CB	0.770	±	0.049	0.744	±	0.046
0.40	RF	0.716	±	0.059	0.700	±	0.057
	XGB	0.724	±	0.026	0.711	±	0.015
	LGBM	0.715	±	0.049	0.728	±	0.050
	CB	0.719	±	0.048	0.678	±	0.050
0.45	RF	0.724	±	0.132	0.711	±	0.119
	XGB	0.733	±	0.094	0.672	±	0.069
	LGBM	0.709	±	0.111	0.678	±	0.085
	CB	0.754	±	0.097	0.650	±	0.061
0.50	RF	0.739	±	0.083	0.650	±	0.075
	XGB	0.702	±	0.059	0.583	±	0.059
	LGBM	0.702	±	0.067	0.622	±	0.082
	CB	0.744	±	0.067	0.633	±	0.080
0.55	RF	0.713	±	0.059	0.644	±	0.057
	XGB	0.737	±	0.063	0.656	±	0.025
	LGBM	0.728	±	0.071	0.656	±	0.064
	CB	0.769	±	0.080	0.683	±	0.097
0.40PMT	RF	0.452	±	0.105	0.522	±	0.077
	XGB	0.531	±	0.066	0.578	±	0.060
	LGBM	0.467	±	0.031	0.489	±	0.032
	CB	0.467	±	0.048	0.533	±	0.063

MLs, RF, XGB, LGBM, and CB indicated machine learnings, random forest, eXtreme Gradient Boosting, Light Gradient Boosting Machine, and Catboost, respectively. Each average and standard deviation (SD) were calculated by 5-fold cross-validation. Most high-performance of prediction in each MAX Scores were indicated by bold. 0.40PMT showed permutation test in 0.40 of MAX Score.

## Supplementary Materials

The following are available online, Figure S1: Prediction performance at different snapshot angles by DeepSnap, Figure S2: Differences in the mean levels of performance of DeepSnap at different angles, Figure S3: Data split, Table S1a: Optimization of the solver types in hyperparameters, Table S1b: Optimization of the Learning rates and Batch sizes in hyperparameters with Loss (Val), Table S1c: Optimization of the Learning rates and Batch sizes in hyperparameters with Acc (Val), Table S2: Performance of prediction models with thresholds of Max score, Table S3a: Clustering analysis of principal components of molecular descriptors extracted by MORDRED from training dataset, Table S3b: Clustering analysis of principal components of molecular descriptors extracted by MORDRED from Test datasets, Table S4: Chemical compound and MAX value used in this study.

## Abbreviations

2D: two-dimensional
3D: three-dimensional
Acc (Test): accuracy in the test dataset
AhR: aryl hydrocarbon receptor
AO: adverse outcome
AOP: adverse outcome pathway
AUC: area under the curve
Acc (Val): accuracy in the validation dataset
BAC: balanced accuracy
BSs: batch sizes
CB: CatBoost
CNN: convolutional neural network
DIGITS: deep learning GPU training system
DL: deep learning
DModX: distance to the model in X space
DNNs: deep neural networks
DILI: drug-induced liver injury
F: F value
FN: false negative
FP: false positive
LGBM: light gradient boosting machine
LR: learning rate
Loss (Val): loss in the validation dataset
MAX value: maximum fold-change value of AhR reporter activity
MCC: Matthews correlation coefficient
MDCs: metabolism-disrupting chemicals
MIE: molecular initiating event
ML: machine learning
MOE: molecular operating environment
PCA: principle component analysis
RF: random forest
ROC: receiver operating characteristic
SMILES: simplified molecular input line entry system
TN: true negative
TP: true positive
XGB: eXtreme gradient boosting
XRE: xenobiotic-response element
