# Supplementary material for: Prediction Model of Aryl Hydrocarbon Receptor Activation by a Novel QSAR Approach, DeepSnap–Deep Learning
Source: Molecules. 2020 Mar 13;25(6):1317. doi: 10.3390/molecules25061317 (PMC7144728; doi:10.3390/molecules25061317)
Supplement: Supplementary file 1 [file molecules-25-01317-s001.pdf]

Fig.S1

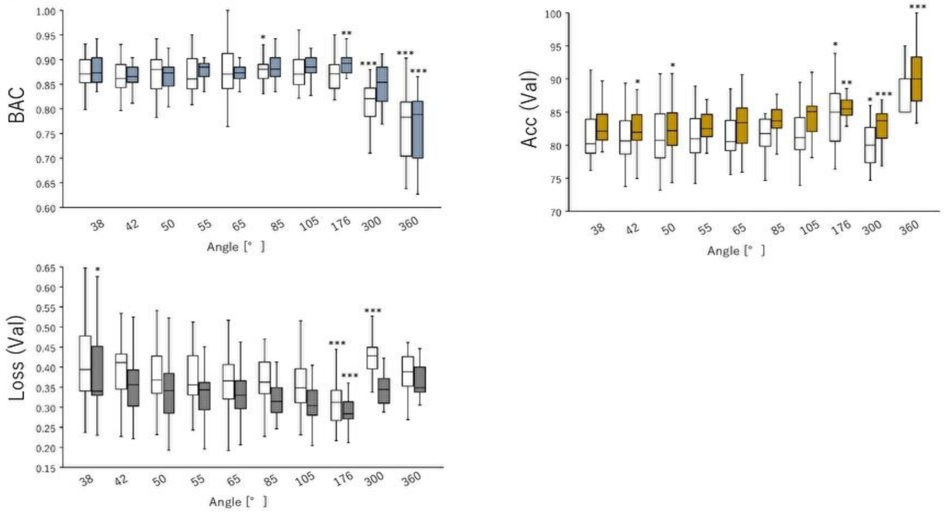

Figure S1. Prediction performance at different snapshot angles by DeepSnap. For each of the 10 angles (38°, 42°50°, 55°, 65°, 85°, 105°, 176°, 300°, 360°), an unfilled box (left) and a filled box (right) indicate datasets ratios Tra:Val:Test = 1:1:1 and 2:2:1, respectively. The means of MMC, F, Acc (Test), AUC, Acc (Val), Loss (Val), and BAC were calculated by 25-fold. * : $p < .05$, * *: $p < .01$, ** *: $p < .001$.

Fig.S2

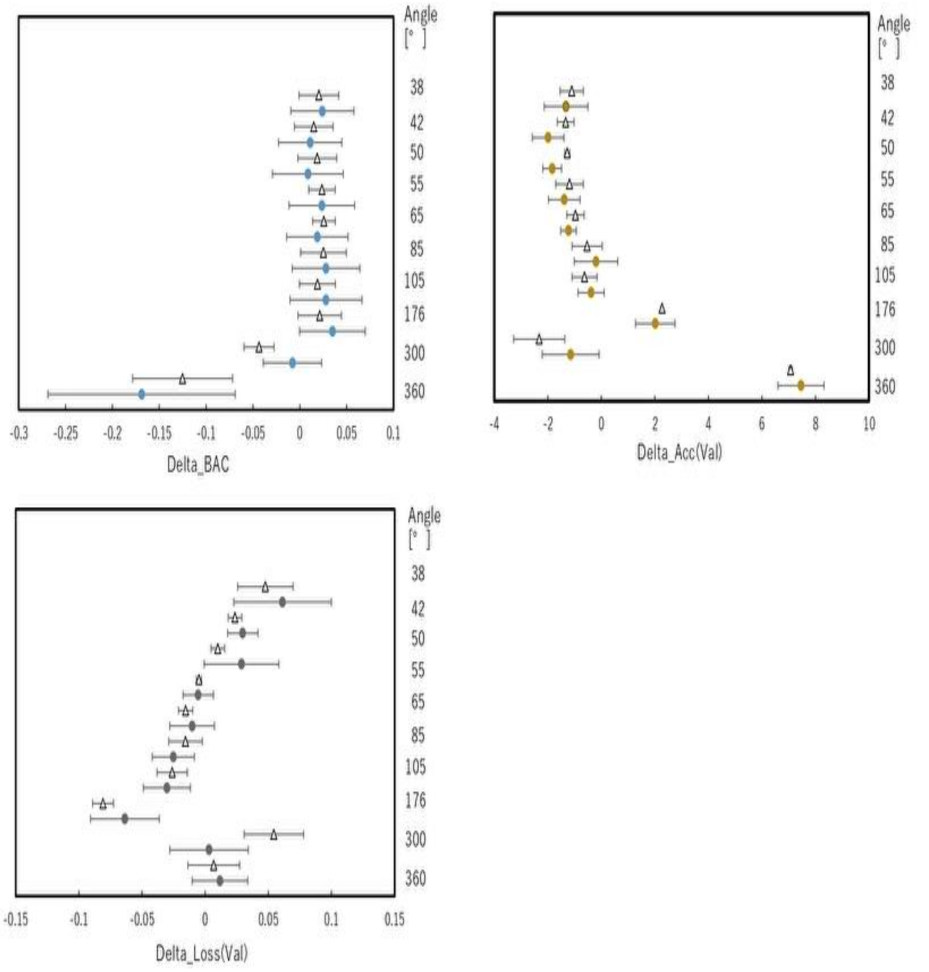

Figure S2. Differences in the mean levels of performance of DeepSnap at different angles. Differences in the mean values of the performance indicators between one angle and the other nine angles, denoted as Delta_ Acc (Val), Delta_Loss (Val), and Delta_BAC, were calculated based on the results shown in Fig. S1, with a 95% confident interval (CI) as error bars. The filled dots and unfilled triangles indicate the dataset ratios Tra: Val: Test = 1: 1: 1 and 2: 2: 1, respectively.

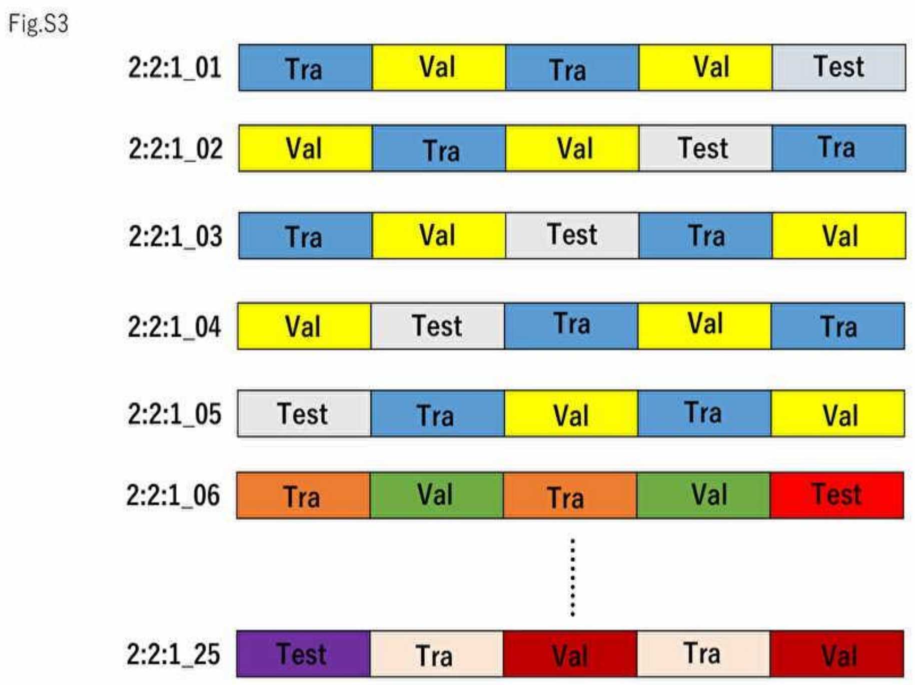

Figure S3. Preparation of dataset split into Tra, Val, and Test. Three dataset groups, including Tra, Val, and Test, were then built with a ratio of 2:2:1. A prediction model was created by the Tra ad Val datasets, respectively. Finally, prediction performance was calculated by using the Test dataset (2:2:1_01). Finally, a total of 25 tests were performed (2:2:1_25).

Table S1a. Optimization of the solver types in hyperparameters

Solver Type	Loss (Val)	Acc (Val)
NAG	**0.259221**	87.1231
AdaGrad	0.273683	83.7705
AdaDelta	0.410498	80.9836
Adam	0.344194	85.5738
RMSprop	0.317712	**87.7049**
SDG	0.313697	83.9753

Angle:176° , BT:40, LR:0.001

Most high-performance of each Solver types were indicated by bold.

Table S1b. Optimization of the Learning rates and Batch sizes in hyperparameters with Loss (Val)

	LRs	LRs	LRs	LRs	LRs	LRs	LRs	LRs
BSs	0.0025	0.0024	0.0023	0.0022	0.0021	0.0020	0.0019	0.0018
35	0.187423	0.182966	0.189255	0.187056	0.183283	0.187059	0.178084	0.186253
36	0.177527	0.189400	0.187449	0.185641	0.184379	0.186366	0.188569	0.190117
37	**0.146634**	0.154745	0.153164	0.154488	0.154003	0.153302	0.154567	0.152418
38	0.171144	0.171241	0.170632	0.171688	0.174109	0.173029	0.173709	0.171152
39	0.155759	0.159271	0.157051	0.156157	0.158705	0.159942	0.159590	0.158212
40	0.164046	0.167822	0.168609	0.173596	0.172465	0.170931	0.171160	0.168201

LRs: learning rates, BSs:Batch sizes
Angle:176° , Solver type: NAG
Most high-performance of each predictions were indicated by bold.

Table S1c. Optimization of the Learning rates and Batch sizes in hyperparameters with Acc (Val)

	LRs	LRs	LRs	LRs	LRs	LRs	LRs	LRs
BSs	0.0025	0.0024	0.0023	0.0022	0.0021	0.0020	0.0019	0.0018
35	91.8804	91.4529	90.5983	91.2393	91.0257	88.2479	91.2393	91.4529
36	91.7211	91.0676	91.2854	91.0676	90.6318	90.4140	90.6318	90.6318
37	**94.0816**	92.4490	93.2653	92.8572	93.0612	92.6531	92.6531	92.6531
38	92.2917	92.2917	92.2917	92.2917	92.2917	90.8333	91.4583	91.4583
39	92.5532	92.1277	91.7021	92.1277	92.3404	91.0638	91.9149	92.3404
40	92.8261	92.8261	92.0913	92.6087	91.0869	92.8261	91.9565	93.2609

LRs: learning rates, BSs:Batch sizes
Angle:176° , Solver type: NAG
Most high-performance of each predictions were indicated by bold.

Table S2. Performance of prediction models with thresholds of Max score.

Max Score	average Loss(Val) ± SD			average Acc(Val) ± SD			average BAC ± SD			average F ± SD		
0.10	0.221	±	0.050	93.453	±	1.782	0.878	±	0.096	0.662	±	0.139
0.15	0.301	±	0.065	88.561	±	2.232	0.804	±	0.077	0.541	±	0.143
0.20	0.342	±	0.049	82.284	±	2.869	0.851	±	0.056	0.704	±	0.065
0.30	0.325	±	0.029	82.272	±	2.645	0.903	±	0.036	0.849	±	0.033
0.35	0.279	±	0.030	86.756	±	1.611	0.903	±	0.051	0.848	±	0.044
0.40	0.232	±	0.056	89.638	±	3.012	**0.933**	±	0.040	0.904	±	0.041
0.45	0.287	±	0.095	89.489	±	3.230	0.890	±	0.052	0.877	±	0.037
0.50	**0.142**	±	0.063	**95.560**	±	2.455	0.921	±	0.010	**0.914**	±	0.027
0.55	0.195	±	0.061	93.433	±	2.019	0.858	±	0.070	0.871	±	0.071
0.40PMT	0.666	±	0.013	61.431	±	4.672	0.594	±	0.107	0.469	±	0.215

Each average and standard deviation (SD) were calculated by 5-fold.
Most high-performance of prediction in each Max Scores were indicated by bold.
0.40PMT showed permutation test in 0.40 of Max Score.

Table S3a. Clustering analysis of principal components of molecular descriptors extracted by MORDRED from training dataset.

	CV01_Tra				CV04_Tra				CV13_Tra				CV17_Tra				CV24_Tra			
			Fluctuation				Fluctuation				Fluctuation				Fluctuation				Fluctuation	
CL No.	variables No.	variables	Cluster	Total	variables No	variables	Cluster	Total	variables No	variables	Cluster	Total	variables No	variables	Cluster	Total	variables No	variables	Cluster	Total
1	63	VR3_D	0.978	0.061	65	VR3_D	0.966	0.061	108	LogEE_A	0.888	0.092	68	nBonds	0.949	0.067	62	VR3_Dzse	0.982	0.059
2	63	nBonds	0.938	0.058	66	VR1_Dzp	0.936	0.06	92	nAtom	0.858	0.076	66	VR3_D	0.969	0.066	61	SpDiam_Dzse	0.949	0.056
3	60	SpDiam_Dzse	0.976	0.058	59	SpMax_Dzse	0.951	0.055	78	SpMax_Dzse	0.909	0.068	63	SpMax_Dzse	0.959	0.062	45	VR1_D	0.961	0.042
4	51	ATS9d	0.962	0.048	44	Si	0.972	0.42	66	LogEE_Dt	0.841	0.053	48	VR1_Dzi	0.96	0.048	44	Spe	0.958	0.041
5	31	MPC5	0.903	0.028	32	ATS1v	0.933	0.029	41	Sm	0.805	0.032	26	ATS7v	0.935	0.025	34	ATS5d	0.941	0.031
6	28	ATS1Z	0.902	0.025	24	AATS0se	0.925	0.022	37	AATS1se	0.824	0.029	26	MPC5	0.903	0.024	26	AATS0se	0.904	0.023
7	27	Mare	0.898	0.024	25	Xp-7d	0.885	0.022	37	nBondsM	0.726	0.026	24	Mse	0.902	0.022	22	Sm	0.886	0.019
8	23	ATS7d	0.934	0.021	22	SM1_Dzp	0.904	0.019	36	SM1_Dzare	0.742	0.026	22	MWC10	0.917	0.021	19	ATS7p	0.909	0.017
9	23	SM1_Dzare	0.896	0.02	18	Xpc-4d	0.869	0.015	35	Mm	0.719	0.024	21	SM1_Dzare	0.852	0.018	17	Xp-5d	0.899	0.015
10	18	ATS4dv	0.885	0.016	16	ATS2z	0.91	0.014	27	MATS2are	0.635	0.016	16	SZ	0.897	0.015	17	nAromAtom	0.871	0.014
means	38.7		0.927	0.036	37.1		0.925	0.072	55.7		0.795	0.044	38.0		0.924	0.037	34.7		0.926	0.032
sd	18.3		0.035	0.018	19.8		0.034	0.124	28.3		0.087	0.026	20.9		0.037	0.021	17.4		0.037	0.017
total	387			0.359	371			0.717	557			0.442	380			0.368	347			0.317

CL No. : cluster number that is top 10 of clusters of percentage of total variation explained by each cluster component

variables No. : variables number that is number of variables belonging to each cluster

variables: representative variable with the largest square of correlation coefficient with cluster component

Fluctuation in Cluster: the percentage of fluctuation explained by their first PC of the fluctuations of variables belonging to the cluster

Fluctuation in Total: percentage of total variation explained by each cluster component

Table S3b. Clustering analysis of principal components of molecular descriptors extracted by MORDRED from Test datasets.

		CV01_Test				CV04_Test				CV13_Test				CV17_Test				CV24_Test			
				Fluctuation				Fluctuation				Fluctuation				Fluctuation				Fluctuation	
CL No.	variables No.	variables No.	variables	Cluster	Total	variables No	variables	Cluster	Total	variables No	variables	Cluster	Total	variables No	variables	Cluster	Total	variables No	variables	Cluster	Total
1	63	72	ATS6se	0.964	0.072	65	VR3_DzZ	0.968	0.063	91	LogEE_Dzpe	0.961	0.086	62	VR3_Dzse	0.982	0.059	79	Sse	0.964	0.076
2	63	66	VR3_D	0.954	0.065	55	LogEE_Dzi	0.975	0.054	84	nHeavyAtom	0.967	0.08	61	SpDiam_Dzse	0.949	0.056	76	LogEE_Dzse	0.973	0.074
3	60	62	MID	0.946	0.061	49	VR1_D	0.963	0.047	60	Sse	0.966	0.057	45	VR1_D	0.961	0.042	58	VR3_Dzse	0.957	0.055
4	51	52	LogEE_Dzi	0.961	0.052	37	ATS2p	0.958	0.036	56	LogEE_A	0.974	0.054	44	Spe	0.958	0.041	52	VR1_Dzse	0.957	0.05
5	31	29	SM1_Dzare	0.878	0.026	36	ATS0i	0.956	0.035	29	SM1_Dzare	0.913	0.026	34	ATS5d	0.941	0.031	48	Zagreb1	0.963	0.046
6	28	26	MPC5	0.888	0.024	34	ATS7p	0.927	0.032	28	MPC2	0.927	0.026	26	AATS0se	0.904	0.023	32	SM1_Dzare	0.914	0.029
7	27	24	MW	0.887	0.022	26	TMPC10	0.909	0.026	23	MPC5	0.984	0.02	22	Sm	0.886	0.019	23	AATS0se	0.925	0.021
8	23	18	AATS0pe	0.931	0.017	24	ATS2Z	0.923	0.022	21	AATS0pe	0.907	0.019	19	ATS7p	0.909	0.017	17	AATS2p	0.833	0.014
9	23	17	MWC09	0.932	0.016	25	AATSC0pe	0.85	0.021	21	MWC10	0.896	0.019	17	Xp-5d	0.899	0.015	15	MWC10	0.935	0.014
10	18	18	nAromAtom	0.835	0.016	24	AATS0pe	0.866	0.021	17	nAromAtom	0.853	0.014	17	nAromAtom	0.871	0.014	15	TMPC10	0.899	0.013
means	38.7	38.4		0.918	0.037	37.5		0.930	0.036	43.0		0.935	0.040	34.7		0.926	0.032	41.5		0.932	0.039
sd	18.3	22.0		0.043	0.023	14.4		0.043	0.015	27.7		0.042	0.027	17.4		0.037	0.017	24.6		0.042	0.024
total	387	384			0.371	375			0.357	430			0.401	347			0.317	415			0.392

CL No. : cluster number that is top 10 of clusters of percentage of total variation explained by each cluster component

variables No. : variables number that is number of variables belonging to each cluster

variables: representative variable with the largest square of correlation coefficient with cluster component

Fluctuation in Cluster: the percentage of fluctuation explained by their first PC of the fluctuations of variables belonging to the cluster

Fluctuation in Total: percentage of total variation explained by each cluster component

Table S4. Chemical compounds used in this study

#	MAX	SMILES
1	1.290358273	CC(=C)C(=O)OCC[N+](C)(C)C
2	1.025980472	BrCCCCCCCCCCBr
3	1.214630182	Cc1c(C)cccc1C
4	1.239914837	CCCCC(COC(=O)c1cc(ccc1C(=O)OCC(CCCC)CC)C(=O)OCC(CCCC)CC)CC
5	1.130144827	Cc1ccc(c(c1)C)C
6	1.158769767	ClCCOCCOCCCl
7	1.228220876	ClCc1ccc(c(c1)Cl)Cl
8	0.555470775	Clc1ccccc1Cl
9	1.207688177	Oc1cc(O)cc(c1)O
10	1.346182263	C(C=C)N1C(=O)N(C(=O)N(C1=O)CC=C)CC=C
11	0.90143508	NCc1cccc(c1)CN
12	0.895002535	BrCCCBr
13	0.970142321	N#Cc1cccc(c1)C#N
14	0.883608263	O=C1OCCO1
15	2.291864268	N=C(Nc1ccccc1)Nc1ccccc1
16	11.21037774	c1ccc(cc1)Nc1ccc(cc1)Nc1ccccc1
17	0.836701879	BrCC(=O)OCC=CCOC(=O)CBr
18	1.194246899	Brc1ccc(cc1)Br
19	0.937811841	ClCCCCCCCCCCl
20	1.092444621	ClCCCBr
21	1.041661001	ClCCc1ccccc1
22	1.065875182	Clc1cccc2c1cccc2
23	0.999108981	COc1cccc2c1cccc2
24	2.895204504	CCNc1cccc2c1cccc2
25	0.89470894	Oc1ccc(c2c1cccc2)S(=O)(=O)O
26	1.497026959	OC(=O)Cc1cccc2c1cccc2
27	1.622967353	CCCCCCCCO[C@@H]1O[C@H](CO)[C@H]([C@@H]([C@H]1O)O)O
28	1.12655414	ClC(C(Cl)(Cl)Cl)COCC(C(Cl)(Cl)Cl)Cl
29	1.798920806	Clc1c(ccc(c1Cl)N)Cc1ccc(c(c1Cl)Cl)N
30	1.416514696	CC(c1cc(Cc2cc(c(c(c2)C(C)(C)C)O)C(C)(C)C)cc(c1O)C(C)(C)C)(C)C
31	1.608267637	Cc1cc(Cc2cc(C)cc(c2O)C(C)(C)C)c(c(c1)C(C)(C)C)O
32	1.285114155	CC(CC(CC(C)(C)C)(C)C)CC(C)(C)C
33	1.290573684	ClC1=C(Cl)C(=O)C(=C(C1=O)Cl)Cl
34	0.968139328	Cc1ccc(c(c1O)C)C
35	1.004008481	BrC(C(C(=O)O)Br)C(=O)O
36	1.405778255	Cc1c(C)cccc1N
37	0.64847916	[O-][N+](=O)c1cc([N+](=O)[O-])c(c(c1)[N+](=O)[O-])O
38	1.232989407	Oc1ccc(cc1C(C)(C)C)C(C)(C)C
39	1.080082864	Brc1ccc(c(c1)Br)O
40	3.464665256	Cc1ccc(c(c1)C)N
41	1.157935847	[O-][N+](=O)c1ccc(c(c1)[N+](=O)[O-])O
42	1.833100568	Cc1ccc(c(c1)N)C
43	1.741676548	CCc1cc(c(c(c1)C(C)(C)C)O)C(C)(C)C
44	0.831977735	OCCOC(C)C
45	1.044843263	CCCCN(CCCC)CCO
46	1.074022645	Cc1cc(N)c(cc1Cl)S(=O)(=O)O
47	1.061049414	Cc1ccc(c(c1)S(=O)(=O)O)N
48	0.936003679	OCC([N+](=O)[O-])(CO)Br
49	1.276519284	CC(=NO)CC
50	1.523412931	CCCCOCCOP(=O)(OCCOCCCC)OCCOCCCC
51	1.48195285	OC(=O)c1ccccc1Cl
52	1.48195285	OC(=O)c1ccccc1Cl
53	1.045172982	Oc1ccccc1Cl
54	3.214101381	CCc1ccc2c(c1)C(=O)c1c(C2=O)cccc1
55	1.012893948	CCCCC(COP(=O)(Oc1ccccc1)Oc1ccccc1)CC
56	1.150123689	CCCCC(COC=C)CC
57	0.456235456	CCCCCCCCOc1ccc(c(c1)O)C(=O)c1ccccc1
58	1.019175031	S=C1NCCN1
59	1.137350701	CC(COc1ccc2c(c1)cccc2)C
60	1.501256801	SC=1NC2=C(N1)C=CC=C2
61	1.501256801	SC=1NC2=C(N1)C=CC=C2
62	2.273356451	Sc1ccc2c(c1)cccc2
63	1.243071436	Cc1ccc(c(c1)[N+](=O)[O-])O
64	0.702123207	C=CCN(CC=C)CC=C
65	1.020354267	C=CC(=O)N(C)C
66	0.715433772	Oc1ccccc1C(C)(C)C
67	1.018010417	C(=C)C1=NC=CC=C1
68	1.274942118	OC(=O)CCSCCC(=O)O
69	0.66176264	CN(c1ccc(cc1)C1(OC(=O)c2c1ccc(c2)N(C)C)c1ccc(cc1)N(C)C)C
70	0.66176264	CN(c1ccc(cc1)C1(OC(=O)c2c1ccc(c2)N(C)C)c1ccc(cc1)N(C)C)C
71	3.060928819	Nc1ccc(c(c1)C)C
72	1.210005404	Cc1cc(C)cc(c1)N
73	0.888955557	ClC=1N=NC(=CC1)Cl
74	0.916636628	CC(=O)C1CCOC1=O
75	0.759644735	Nc1cccc(c1)S(=O)(=O)O
76	1.405939062	Nc1cccc(c1)O
77	1.405939062	Nc1cccc(c1)O
78	1.374134963	Oc1cccc(c1)Br
79	0.825482117	C(#N)C=1C=NC=CC1
80	1.213709619	OCCC(OC)(C)C
81	1.137122957	COC(CCOC(=O)C)C
82	1.706254545	Cc1cccc(c1)O
83	1.196603987	Nc1cccc(c1)[N+](=O)[O-]
84	1.042530915	OC(=O)c1cccc(c1C(=O)O)[N+](=O)[O-]
85	1.304564521	Cc1cccc(c1)Oc1ccccc1
86	1.759000574	Oc1ccc(cc1)Cc1ccc(cc1)O
87	1.759000574	Oc1ccc(cc1)Cc1ccc(cc1)O
88	2.562425837	NNS(=O)(=O)c1ccc(cc1)Oc1ccc(cc1)S(=O)(=O)NN
89	1.116521978	Oc1ccc(cc1)S(=O)(=O)c1ccc(cc1)O
90	1.750060628	CC(c1cc(Sc2cc(c(cc2C)O)C(C)(C)C)c(cc1O)C)(C)C
91	1.383827165	CC(c1cc(Br)c(c(c1)Br)O)(c1cc(Br)c(c(c1)Br)O)C
92	1.525049249	CC(=C)c1ccc(cc1)O
93	1.62750912	CCC(c1ccc(cc1)O)C
94	0.730542367	Nc1ccc(cc1)O
95	1.505271253	Oc1ccc(c(c1)C)Cl
96	1.30792134	Clc1ccc(c(c1)C)O
97	1.071496961	O=Cc1ccc(cc1)Cl
98	1.808621801	Oc1ccc(cc1)Cl
99	2.426550417	CCOc1ccc(cc1)N
100	1.120702676	CCc1ccc(cc1)c1ccccc1
101	0.905165617	CCN1CCOCC1
102	1.699328541	CCc1ccc(cc1)O
103	1.060218551	OC(=O)c1ccc(cc1)C
104	1.314967874	COc1cc(ccc1N)[N+](=O)[O-]
105	0.991827043	CC(=O)C=Cc1ccccc1
106	2.3780942	C(=C)C1=CC=NC=C1
107	0.95441098	CC=C1CC2CC1C=C2
108	1.268373649	C(CC)C1=CC(NC(N1)=S)=O
109	1.359150368	Nc1ccc2c(c1)cc(cc2O)S(=O)(=O)O
110	4.338139293	c1cc2cccc3c2c(c1)CC3
111	3.63739533	c1cc2cccc3c2c(c1)C=C3
112	0.968084868	CCCCOC(=O)C(OC(=O)C)(CC(=O)OCCCC)CC(=O)OCCCC
113	0.808354718	CCCCOCCOCCOC(=O)CCCCC(=O)OCCOCCOCCCC
114	1.012910494	ClCc1ccc(cc1)[N+](=O)[O-]
115	1.061096104	COc1ccc(c(c1)Cl)OC
116	1.061096104	COc1ccc(c(c1)Cl)OC
117	1.48694761	NS(=O)(=O)c1ccccc1
118	10.74418843	O=C1c2ccccc2c2c3c1cccc3ccc2
119	0.934175706	N#Cc1ccccc1
120	1.020862832	C[N+](Cc1ccccc1)(C)C
121	4.73941103	BrC=Cc1ccccc1
122	3.222750293	Nc1ccccc1c1ccccc1
123	1.257433257	CCCCC(COC(=O)/C=C/C(=O)OCC(CCCC)CC)CC
124	0.958170661	CC=CC(=O)N(c1ccccc1C)CC
125	0.997943036	C1CCC2C(C1)CCCC2
126	1.213882167	c1ccc2c(c1)oc1c2cccc1
127	1.362126578	c1ccc2c(c1)sc1c2cccc1
128	0.912624449	CCCCOC(=O)CCCCC(=O)OCCCC
129	0.856558882	CC(c1ccccc1)(OOC(c1ccccc1)(C)C)C
130	1.079031759	C1CCC(CC1)NC1CCCCC1
131	2.521566015	CCCCCCCOC(=O)c1ccccc1C(=O)OCCCCCCC
132	0.37166227	[O-][N+](=O)c1cc(ccc1Nc1ccccc1)S(=O)(=O)Nc1ccccc1
133	1.181697207	CCCCCCCCCCCCN(C)C
134	1.152248643	CCOC(=O)N
135	0.997041894	CCC1CCCCC1
136	0.99259339	C#C[C@]1(O)CC[C@@H]2[C@]1(C)CC[C@H]1[C@H]2CCc2c1ccc(c2)O
137	0.979919813	CCCC[C@H]([C@H]([C@@H](OC(=O)C[C@H](C(=O)O)CC(=O)O)C[C@H](C[C@@H](CCCC[C@H](C[C@@H]([C@@H](N)C)O)O)O)C)OC(=O)C[C@H](C(=O)O)CC(=O)O)C
138	3.685771184	N=C(Nc1ccccc1C)Nc1ccccc1C
139	1.095564173	CC(OOC(C)(C)C)(CCC(OOC(C)(C)C)(C)C)C
140	0.734709793	O=C1NCC(=O)N1
141	1.457855839	CN(c1ccc(cc1)C(c1ccc(cc1)N(C)C)c1ccccc1)C
142	1.150317082	CCc1cccc(c1)O
143	0.942753372	CC(=C)C(=O)N
144	1.058223239	CC(=C)C#N
145	1.058223239	CC(=C)C#N
146	1.087162625	CC1CCCCC1
147	1.087162625	CC1CCCCC1
148	1.258083122	Nc1cc(cc2c1c(O)cc(c2)S(=O)(=O)O)S(=O)(=O)O
149	1.113750581	O=C(Nc1ccc(cc1C)c1ccc(c(c1)C)NC(=O)CC(=O)C)CC(=O)C
150	0.842463307	CCN(C(=O)c1cccc(c1)C)CC
151	0.842463307	CCN(C(=O)c1cccc(c1)C)CC
152	1.162741671	CCN(CC)O
153	1.102946784	CN(Cc1ccccc1)C
154	1.00100081	CC(CC(Nc1ccc(cc1)Nc1ccccc1)C)C
155	0.938394524	NCCNCCO
156	3.114282404	C1(CCCCC1)NSC=1SC2=C(N1)C=CC=C2
157	1.266869173	CCNc1ccccc1
158	0.864588909	CCCCCCCCCCCCCCCC
159	1.151902846	CN(c1ccccc1)C(=O)C
160	1.103349729	CNc1ccccc1
161	1.552914567	CCCCCCCCCCCCCCC
162	1.878193966	CC(Nc1ccc(cc1)Nc1ccccc1)C
163	10.83896477	O=C(c1cc2ccccc2cc1O)Nc1ccccc1
164	10.83896477	O=C(c1cc2ccccc2cc1O)Nc1ccccc1
165	1.070129058	[O-][N+](=O)c1ccccc1
166	1.070129058	[O-][N+](=O)c1ccccc1
167	1.458956088	Cc1ccccc1S(=O)(=O)N
168	1.458956088	Cc1ccccc1S(=O)(=O)N
169	1.520742094	Oc1ccc(cc1)C(c1ccccc1)(C)C
170	1.346967104	Oc1ccc(cc1)[N+](=O)[O-]
171	0.909585979	Cc1ccc(cc1)C(C)(C)C
172	1.140770057	CC(c1ccc(cc1)O)(CC(C)(C)C)C
173	1.080578756	CC(=O)Nc1ccc(cc1)S(=O)(=O)Cl
174	1.288789516	CC1OC(C)OC(O1)C
175	4.748249663	Clc1c(O)c(Cl)c(c(c1Cl)Cl)Cl
176	1.072274531	CCC1(C(=O)NC(=NC1=O)O)c1ccccc1
177	1.191077131	CCC(c1cc(c(c(c1)C(C)(C)C)O)C(C)(C)C)C
178	1.777203605	CC(c1ccc(cc1)O)(c1ccc(cc1)O)C
179	1.292010615	NC(=O)Nc1ccccc1
180	1.003750074	Oc1ccc2c(c1)c(cc(c2)S(=O)(=O)O)S(=O)(=O)O
181	1.775748111	Nc1c2C(=O)c3ccccc3C(=O)c2c(cc1S(=O)(=O)O)Nc1cccc(c1)S(=O)(=O)CCOS(=O)(=O)O
182	0.993195258	Oc1cc2ccc(cc2cc1S(=O)(=O)O)S(=O)(=O)O
183	0.98112367	[O-][N+](=O)c1cccc(c1)S(=O)(=O)O
184	1.22829331	Nc1ccc(c2c1cccc2)S(=O)(=O)O
185	0.968677068	C=Cc1ccc(cc1)S(=O)(=O)O
186	1.014127265	Cc1ccc(cc1)S(=O)(=O)O
187	1.232350555	Nc1ccc(cc1)S(=O)(=O)N
188	0.872096485	N#Cc1ccc(cc1)C#N
189	0.89889518	CC(=C)C(=O)OC(C)(C)C
190	1.161769994	CCC(c1ccccc1)(C)C
191	0.817606598	BrC(C(Br)Br)Br
192	0.91190244	OCC1CCCO1
193	1.030273433	O=S1(=O)CCCC1
194	1.40950012	C1C2CC3CC1CC(C2)C3
195	1.585998727	COP(OC)OC
196	1.102820213	CCCCC(COP(=O)(OCC(CCCC)CC)OCC(CCCC)CC)CC
197	1.487257664	ClC(c1ccccc1)(c1ccccc1)c1ccccc1
198	0.775277916	C=CC1(C)OC(=O)N(C1=O)c1cc(Cl)cc(c1)Cl
199	1.268373649	N1C(=S)NC(=O)C=C1CCC
200	0.99259339	C#C[C@]1(O)CC[C@@H]2[C@]1(C)CC[C@H]1[C@H]2CCc2c1ccc(c2)O
201	1.750060628	CC(c1cc(Sc2cc(c(cc2C)O)C(C)(C)C)c(cc1O)C)(C)C
